# Association between the *CYP4A11* T8590C Variant and Essential Hypertension: New Data from Han Chinese and a Meta-Analysis

**DOI:** 10.1371/journal.pone.0080072

**Published:** 2013-11-21

**Authors:** Hua-Cheng Yan, Jun-Hua Liu, Jian Li, Bao-Xia He, Liang Yang, Jian Qiu, Liang Li, Da-Peng Ding, Lei Shi, Shu-Jin Zhao

**Affiliations:** 1 Department of Pharmacy, Guangzhou General Hospital of Guangzhou Military Command, Guangzhou, China; 2 Department of Cardiology, Guangzhou General Hospital of Guangzhou Military Command, Guangzhou, China; 3 Department of Pharmacy, Henan Cancer Hospital, Zhengzhou, China; 4 School of Basic Medical Sciences, Southern Medical University, Guangzhou, China; 5 Institute of Genetic Engineering, Southern Medical University, Guangzhou, China; Morehouse School of Medicine, United States of America

## Abstract

**Objective:**

CYP4A11 oxidizes endogenous arachidonic acid to 20-hydroxyeicosatetraenoic acid, a renal vasoconstrictor and natriuretic in humans. Previous studies demonstrated an association between a functional variant (T8590C) of *CYP4A11* and essential hypertension, though with conflicting results. To elucidate this relationship, a case-control study and meta-analysis were performed to assess the possible association of essential hypertension with *CYP4A11* genetic variations.

**Methods:**

Associations between the T8590C polymorphism and essential hypertension were examined in 328 unrelated cases and 297 age-matched controls in Han Chinese individuals. High-resolution melting was used to identify the *CYP4A11* variant. To further investigate the association, we conducted a meta-analysis including eight studies published previously in July 2012.

**Results:**

The frequency of the *CYP4A11* T8590C polymorphism showed no significant difference between cases and controls (all *P*>0.05). However, the meta-analysis showed that the *CYP4A11* T8590C polymorphism may increase the risk of essential hypertension in an additive model (OR: 1.15, 95% CI: 1.02–1.29, *P* = 0.02), a dominant model (OR: 1.06, 95% CI: 1.01–1.32, *P* = 0.03), a recessive model (OR: 1.52, 95% CI: 1.15–2.02, *P* = 0.003) and a homozygote contrast (OR: 1.38, 95% CI: 1.07–1.78, *P* = 0.01). Also, a significant relationship was observed among Caucasians in the additive model, the homozygote contrast, the recessive model and the dominant model (all *P*<0.05). However, no association was observed in an Asian population (all *P*>0.05).

**Conclusions:**

This meta-analysis suggests there is a significant association between the *CYP4A11* T8590C variant and essential hypertension, especially in Caucasians. The case-control study did not find a significant association among the Han Chinese population, but the controls were poorly matched and meaningful conclusions cannot therefore be made. Further large-scale studies are needed to clarify whether the *CYP4A11* T8590C polymorphism is associated with hypertension risk in Asians or has a gender-specific effect.

## Introduction

Hypertension is a major risk factor for cardiac, renal and cerebrovascular morbidity and mortality [1]. It is important to understand individual susceptibility to essential hypertension by the use of molecular and genetic approaches, with a view to delivering targeted or individualized therapies to improve the survival rate and quality of life of hypertensive patients.

Hypertension has a substantial heritability that is likely to be of polygenic origin. To date, several variants have been identified by genome-wide association studies (GWASs) to be associated with essential hypertension [2], [3]. Other common gene variants have been shown to contribute to essential hypertension according to gender or ethnicity [4]–[6]. Thus, the identification of polygenic determinants is not straightforward.

Cytochrome P450 (CYP) is a superfamily of cysteine-heme enzymes that are key mediators of the oxidative transformation of endogenous and exogenous molecules. Cytochrome P450 enzymes are classified into families, subfamilies and individual isoenzymes based on similarities in their amino acid sequences. Human CYP4A11, which belongs to the cytochrome P450 family 4, catalyzes the conversion of endogenous arachidonic acid to 20-tetrahydrocannabinol acid (20-HETE) [7]; 20-HETE is normally produced in renal and cerebral arterioles, glomeruli and the renal tubules. Experimental models of hypertension indicate that 20-HETE can act in either a pro- or an antihypertensive manner, depending on its site-specific expression in the renal vasculature or tubules, respectively. In the renal tubules, 20-HETE inhibits tubular sodium reabsorption, resulting in natriuresis, and acts as an antihypertensive; whereas, 20-HETE acts as a vasoconstrictor and is pro-hypertensive in the renal vasculature [8].

Given the role of 20-HETE in the regulation of hypertension, CYP4A11 is the subject of much research interest. The human *CYP4A11* gene is located on chromosome 1p33, spans approximately 12.57 kilobase-pairs and contains 12 exons. More than 120 single nucleotide polymorphisms (SNPs) of the human *CYP4A11* gene are listed in the National Center for Biotechnology Information SNP database (http://www.bianca.nl.NIH.gov/SNP). One of the coding SNPs, rs1126742 (T8590C), which results in a phenylalanine to serine substitution at amino acid 434 in exon 11 of CYP4A11, has been found to be associated with blood pressure (BP) in several populations [9]–[11]. However, the results are conflicting and whether the *CYP4A11* T8590C variant has a role in modulating BP in the Chinese Han population remains unknown. Given this background, the aim of this study was to investigate the possible association between the *CYP4A11* T8590C variant and essential hypertension in the Chinese Han population. In addition, a meta-analysis was conducted, including studies examining the association between the *CYP4A11* T8590C variant and the risk of hypertension, to clarify previous inconsistencies.

## Methods

### Subjects

Patients diagnosed with essential hypertension were recruited at Guangzhou General Hospital of Guangzhou Military Command from 2008 to 2011. Three hundred and twenty-eight essential hypertension patients and 297 age-matched controls were enrolled in the study. BP was measured twice in each participant while seated, after 5 min of rest. Essential hypertension was diagnosed based on the following criteria: seated systolic BP greater than 140 mmHg or diastolic BP greater than 90 mmHg on three occasions within 2 months after the first BP reading, or current use of antihypertensive agents. Individuals with secondary hypertension, cancer, chronic kidney or hepatic disease, stroke, diabetes mellitus or severe psychiatric illness were excluded. All individuals enrolled were from the Han population in China. Diagnosis of diabetes was based on the history of treatment for hypoglycemia and/or confirmed fasting blood glucose >7.0 mmol/l. Individuals who had smoked ≥10 cigarettes per day for at least 2 years were defined as smokers and those who had drunk ≥210 g alcohol per week for more than 3 years were defined as drinkers.

This study protocol was approved by the Ethics Committee of Guangzhou General Hospital of Guangzhou Military Command. All individuals in this manuscript gave written informed consent for the publication of their case details.

### DNA Extraction and Genotyping

Peripheral venous blood was drawn from each individual. Genomic DNA was extracted from whole blood using the Tiangen DNA blood kit (Pangenetic Biotech Co., Ltd, Beijing, China). The *CYP4A11* T8590C allele was genotyped by high resolution melting. The primers used were as follows: forward: 5′-TGGGTGGCAAG TAGGTGCTGGA-3′; reverse: 5′-TGTTGAGCAGAACCCGGTGCA-3′. PCR reactions were performed in a final volume of 20 µl using Highlighter 480 High Resolution Melting Master (Reference 04909631001; Roche Diagnostics) and 30 ng DNA. The primers and MgCl_2_ were used at 10 µM and 2.5 M, respectively. The PCR program comprised an initial denaturation-activation step at 95°C for 10 min, followed by a 45-cycle program of denaturation at 95°C for 10 s, annealing at 57.8°C for 10 s and elongation at 72°C for 15 s with reading of the fluorescence and a single acquisition mode. The melting program comprised three steps: denaturation at 95°C for 1 min, renaturation at 40°C for 1 min and then melting, with a continuous fluorescence reading from 60 to 90°C at 25 acquisitions per °C.

### Statistical Analysis

Statistical analysis was performed using the SPSS 13.0 software package (SPSS, Chicago, IL, USA). The genotype frequency distribution for each variant was separately tested for Hardy-Weinberg equilibrium (HWE) with a chi-square test in the case and control groups. Differences of continuous variables with a normal distribution (presented as mean ± SD) between the two groups were calculated using the independent-sample *t*-test. Differences of continuous variables departing from the normal distribution, even after transformation, were tested by the Mann-Whitney *U*-test. Odds ratios (ORs) and their 95% confidence intervals (CIs) were calculated to determine associations between the polymorphisms and essential hypertension. Two-tailed *P* values <0.05 were considered statistically significant.

### Meta-analysis

#### Searching

To explore the association between the T8590C polymorphism of *CYP4A11* and essential hypertension in different studies and ethnicities, we performed a meta-analysis of previously published studies (up to July, 2012) concerning the T8590C polymorphism and essential hypertension. Our own data were also included. We systematically searched PubMed, Medline, Embase and the China National Knowledge Infrastructure (CNKI) using the following keywords: “CYP4A11”, “cytochrome P450 4A11”, “polymorphism”, “gene” and “hypertension”. We also retrieved additional studies by hand, searching the bibliographies of original research reports and review articles, and through the Medline option “related articles”. All relevant referenced articles were also investigated.

The inclusion criteria were: (a) evaluation of the *CYP4A11* T8590C polymorphism and the risk of developing hypertension; (b) unrelated case-control studies or cohort studies; (c) availability of the sample size, ORs and 95% CIs or information including genotype frequency of cases and controls that could help in inferring the study results; and (d) presence of non-overlapping data. For duplicate publications, the study with the smaller data set was excluded. Diagnoses of essential hypertension were made in accordance with the diagnostic requirements of the World Health Organization established in 1999. Subjects with systolic BP≥140 mmHg or diastolic BP≥90 mmHg and subjects who had no secondary hypertension were included.

#### Data extraction

Two investigators reviewed and extracted data from all of the eligible publications independently, according to the inclusion and exclusion criteria listed above. All data were collected according to a standard protocol. Studies that were repeated, of poor research quality, did not meet the inclusion criteria or provided little information or insufficient data were excluded. Study quality was assessed using the 9-star Newcastle-Ottawa Scale [12]. Table 1 lists the characteristics of the extracted data, including the name of the first author, publication date, region, study quality, number of genotypes, alleles and numbers of cases and controls.

**Table 1:** Characteristics of studies included in the meta-analysis and distribution of genotypes and allele frequencies of the *CYP4A11* T8590C polymorphism.

					Cases (n)					Controls (n)					
Author	Country	Ethnicity	StudyQuality*	Year	TT	TC	CC	T	C	TT	TC	CC	T	C	HWE(*p*)
Sugimoto K	Japan	Asian	8	2008	325	157	3	807	183	326	153	15	805	183	0.22
Ward NC	Australian	Caucasian	6	2008	115	41	5	271	51	54	19	1	127	21	0.81
Gainer JV(1)	America	Caucasian	7	2005	126	64	5	316	74	152	41	4	345	49	0.80
Gainer JV(2)	America	Caucasian	7	2005	494	166	10	1154	186	678	180	10	1536	200	0.33
Mayer B	Germany	Caucasian	7	2005	481	149	19	1111	187	574	164	10	1312	184	0.31
Mayer B	Germany	Caucasian	7	2006	152	68	8	372	84	250	79	3	579	85	0.56
Fava C	Sweden	Caucasian	7	2008	2924	800	81	6648	962	1665	475	30	3805	535	0.04
Williams JS	America	white/black/Asian/other	6	2011	227	94	11	548	116	109	34	4	252	42	0.51
Current	China	Asian	7	2012	175	132	21	482	174	150	123	24	423	171	0.60

* Study quality was based on the Newcastle-Ottawa Scale (range: 1–9 stars).

#### Statistical analysis

The association between the *CYP4A11* T8590C polymorphism and essential hypertension was compared between the hypertension and control groups by the OR and the corresponding 95% CI. We evaluated the risk using an additive model (C allele versus T allele), a dominant model (TC+CC versus TT), a recessive model (CC versus TT+TC) and the homozygote contrast (comparison of CC versus TT).

The heterogeneity of the studies was tested using the chi-square-based *Q*-test, and was considered statistically significant when *P*<0.10. The inconsistency index *I* ^2^ was also calculated to evaluate the variation caused by the heterogeneity. A high value of *I* ^2^ indicated a higher probability of the presence of heterogeneity (*I* ^2^ = 0–25%, no heterogeneity; *I* ^2^ = 25–50%, moderate heterogeneity; *I* ^2^ = 50–75%, large heterogeneity; and *I* ^2^ = 75–100%, extreme heterogeneity). If there was heterogeneity among the studies (*P*<0.1), a random-effects model was used to estimate the combined OR. Otherwise, the pooled OR was estimated by the fixed-effect model [13]. The significance of the combined OR was determined by the *Z*-test and the significance was set at *P*<0.05. Fisher’s exact test was used to assess the HWE and the significance was set at *P*<0.05. In addition, sensitivity analysis was carried out by including and excluding studies not in HWE. Potential publication bias was estimated using a funnel plot. Egger’s linear regression test was used to evaluate the funnel plot asymmetry on the natural logarithmic scale of the OR (*P*<0.05 was statistically significant). Review Manager 5.1 and STATA 11.0 software were used to perform statistical analysis (Stactometer, College Station, TX).

## Results

### Case-control Study

Baseline characteristics of the study population are shown in Table 2. Age, BMI and levels of triglyceride and high-density lipoprotein cholesterol were balanced between cases and controls. The patients showed significantly higher levels of total cholesterol (*P*<0.001) and low-density lipoprotein cholesterol (*P = *0.005) than the controls. Additionally, there were significantly higher numbers of males (*P* = 0.041) and smokers (*P*<0.001) in the cases compared to the controls.

**Table 2:** Demographic data for the recruited individuals.

Characteristic	Patients(*n* = 328)	Controls(*n* = 297)	*P*
Age (years)	62.66±11.16	63.15±8.41	0.786
Sex (male), n (%)	188 (57.3)	146 (49.2)	0.041
BMI (kg/m^2^)	23.55±2.76	23.47±3.02	0.741
Smoking, n (%)	113 (34.5)	44 (14.8)	0.000
Drinking, n (%)	108 (32.9)	82 (27.6)	0.149
SBP (mmHg)	137.38±14.6	110.87±9.96	0.000
DBP (mmHg)	78.17±9.96	67.30±7.16	0.000
TC (mmol/l)	4.51±1.09	4.06±1.51	0.000
TG (mmol/l)	1.57±1.08	1.66±0.82	0.215
HDL-C (mmol/l)	1.15±0.29	1.18±0.31	0.121
LDL-C (mmol/l)	2.78±0.86	2.58±0.92	0.005

BMI, body mass index; SBP, systolic blood pressure; DBP, diastolic blood pressure; HDL-C, high-density lipoprotein cholesterol; LDL-C, low-density lipoprotein cholesterol; TC, total cholesterol; TG, triglyceride. Age, SBP, DBP, TC, TG, LDL-C and HDL-C (expressed as mean ± standard deviation) were not normally distributed and were analyzed by the Mann-Whitney *U*-test. BMI (expressed as mean ± standard deviation) was normally distributed and was analyzed by Student’s *t*-test. Other data are expressed as frequencies and percentages and were evaluated by the *χ* ^2^-test.

For the total population, the frequencies of the 8590TT, 8590TC and 8590CC genotypes were 52.0%, 40.8% and 7.2%, respectively. These frequencies did not deviate from those predicted by HWE. The allele frequencies of the 8590C allele in cases and controls were 26.5% and 28.8%, respectively. No significant difference in the genotype frequency was observed between the cases and controls (*P* = 0.636), as shown in Table 3. There were also no significant differences between the two groups in the dominant model (*P* = 0.477), the recessive model (*P* = 0.418) and the additive model (*P* = 0.371). The control group was poorly matched to the cases, particularly with regards to other known predictors of hypertension (gender and smoking), which make the results here difficult to interpret.

**Table 3:** Overall genotype distribution of the *CYP4A11* T8590C polymorphism in patients and controls.

		Cases (*n* = 328)	Controls (*n* = 297)	*χ* ^2^	*P*
Genotype	T/T	175 (53.4)	150 (50.5)	0.905	0.636
	T/C	132 (40.2)	123 (41.4)		
	C/C	21 (6.4)	24 (8.1)		
Dominantmodel	TT	175 (53.4)	150 (50.5)	0.507	0.477
	TC+CC	153 (46.6)	147 (49.5)		
Recessivemodel	CC	21 (6.4)	24 (8.1)	0.657	0.418
	TC+TT	307 (93.6)	273 (91.2)		
Additivemodel	T	482 (73.5)	423 (71.2)	0.799	0.371
	C	174 (26.5)	171 (28.8)		

### Meta-analysis

#### Eligible studies/groups

**Figure 1:**
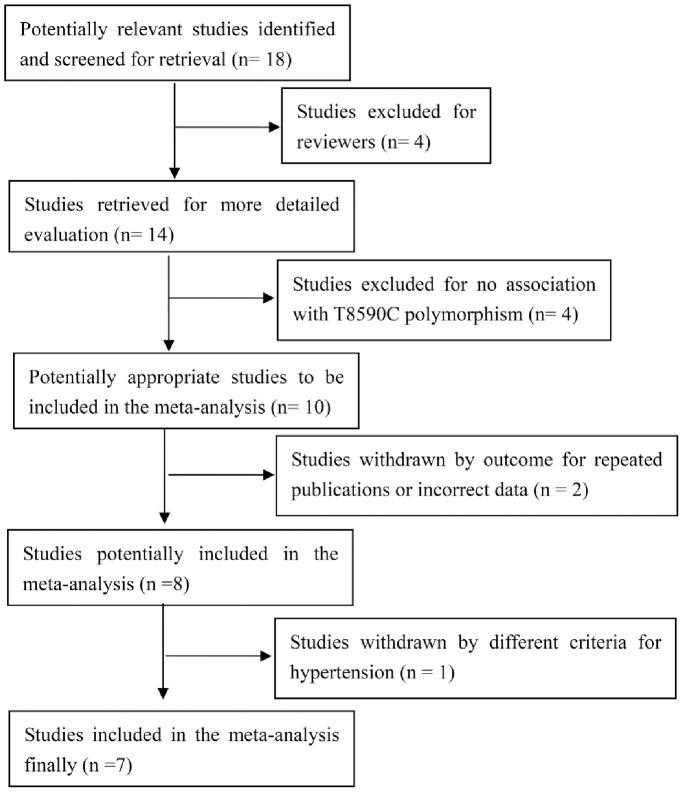
Flow diagram of article selection process for meta-analysis of the *CYP4A11* T8590C polymorphism and hypertension.

Eighteen papers were gathered from the literature research, among which seven papers were eligible based on the study selection criteria. Of the eleven excluded studies, one paper was a repeat publication [10], four were reviews [11], [14]–[16] and four were unrelated to the *CYP4A11* T8590C polymorphism and hypertension [17]–[20]. One paper was excluded because of incorrect data (the combined number in the case group and the control group was not equal to the total number) [21] and one was excluded due to having different criteria for essential hypertension [22] (Figure 1). Finally, data from eight studies (seven previous studies and the present study) were combined [9], [23]–[28], comprising 6863 cases and 5327 controls. Gainer *et al*. [9] reported two independent cohort studies, as shown in Table 1.

#### Characteristics of studies and subjects

The main characteristics of the studies included in our meta-analysis are summarized in Table 1. The ORs differed among the eight studies; some indicated that the presence of the C allele increased the risk of hypertension, whereas the others reported no association between the *CYP4A11* T8590C polymorphism and hypertension risk. Hence, a composite analysis of the study results was performed to draw a reasonable conclusion.

We conducted subgroup analysis classified by ethnicity, gender and HWE population. Five of the studies assessed Caucasian populations, including one from Australia [26], one from the USA [9], one from Sweden [27] and two from Germany [24], [25]. Two studies assessed Asian populations, including one from Japan [23] and one from China. One study included whites, blacks, Asians and others were not analyzed by ethnicity. All studies but one met HWE [27].

Four of the eight eligible studies provided data on male and female subjects. The distribution of genotypes and alleles in males versus females is shown in Table 4. Combined analyses were conducted in the various models.

**Table 4:** *CYP4A11* T8590C genotype frequency and distribution according to gender.

		Cases (n)					Controls (n)					
Author	Gender	TT	TC	CC	T	C	TT	TC	CC	T	C	HWE (*p*)
Gainer JV (1)	Male	66	31	2	163	35	79	18	2	176	22	0.95
	Female	60	33	3	153	39	73	23	2	169	27	0.76
Gainer JV (2)	Male	266	94	7	626	108	316	83	3	715	89	0.40
	Female	228	72	3	528	78	362	97	7	821	111	0.59
Mayer B	Male	274	83	10	631	103	258	85	3	601	91	0.95
	Female	207	66	9	480	84	316	79	7	711	93	0.12
Fava C	Male	1354	356	35	3064	426	604	174	12	1382	198	0.11
	Female	1570	444	46	3584	536	1064	301	18	2429	337	0.18
Current	Male	90	83	15	263	113	80	55	11	215	77	0.78
	Female	85	49	6	219	61	70	68	13	208	94	0.62

#### Main meta-analysis results

The findings for heterogeneity and the combined association between the *CYP4A11* T8590C polymorphism and hypertension are shown in Table 5. Overall, a significant association between the *CYP4A11* T8590C polymorphism and hypertension was found in the additive model (OR: 1.15, 95% CI: 1.02–1.29, *P* = 0.02), the dominant model (OR: 1.06, 95% CI: 1.01–1.32, *P* = 0.03), the recessive model (OR: 1.52, 95% CI: 1.15–2.02, *P* = 0.003) and the homozygote model (OR: 1.38, 95% CI: 1.07–1.78, *P* = 0.01). Seven studies also underwent stratified analysis by ethnicity (one study was excluded because of mixed ethnicity). No increased risk was detected among Asians in the additive model, the homozygote contrast, the recessive model or the dominant model (all *P*>0.05). However, significant differences were detected among Caucasians in the additive model, the homozygote contrast, the recessive model and the dominant model, as shown in Table 5 (all *P*<0.05). Furthermore, four studies that conducted subgroup analysis by gender were pooled and analyzed in subgroups. No statistically significant differences were found for male or female subjects in any of the various statistical models (all *P*>0.05), as shown in Table 6.

**Table 5:** Main odds ratios (ORs) for the *CYP4A11* T8590C polymorphism in the meta-analysis.

Allele and genotype	Populations	OR (95%CI)	I^2^	*P*_heterogeneity_	Analysis model	*P*
C allele vs. T allele	All populations	1.15 (1.02, 1.29)	47%	0.06	Random	0.02
	Caucasians	1.23 (1.06,1.43)	53%	0.06	Random	0.007
	Asians	0.95 (0.80, 1.12)	0	0.52	Fixed	0.54
CC vs. TT	All populations	1.38 (1.07, 1.78)	20%	0.26	Fixed	0.01
	Caucasians	1.74 (1.27, 2.39)	0	0.70	Fixed	0.0006
	Asians	0.80 (0.49, 1.29)	0	0.77	Fixed	0.35
CC+TC vs. TT	All populations	1.06 (1.01, 1.32)	47%	0.06	Random	0.03
	Caucasians	1.23 (1.03, 1.47)	59%	0.03	Random	0.03
	Asians	0.96 (0.79, 1.18)	0	0.54	Fixed	0.71
CC vs. TC+TT	All populations	1.52 (1.15, 2.02)	0	0.59	Fixed	0.003
	Caucasians	1.71 (1.24, 2.34)	0	0.73	Fixed	0.001
	Asians	0.81 (0.50, 1.30)	0	0.84	Fixed	0.38

**Table 6:** Odds ratios (ORs) for the CYP4A11 T8590C polymorphism according to gender.

Allele and genotype	Gender	OR (95% CI)	*I* ^2^	*P*_heterogeneity_	Analysis model	*P*
C allele vs. T allele	Male	1.11 (0.98, 1.26)	40%	0.15	Fixed	0.10
	Female	1.07 (0.84, 1.36)	68%	0.01	Random	0.57
CC vs. TT	Male	1.55 (1.00, 2.41)	0	0.65	Fixed	0.05
	Female	1.33 (0.90, 1.97)	46%	0.11	Fixed	0.16
CC+TC vs. TT	Male	1.18 (0.94, 1.48)	54%	0.07	Random	0.16
	Female	1.07 (0.82, 1.40)	66%	0.02	Random	0.59
CC vs. TC+TT	Male	1.05 (0.40, 2.73)	76%	0.002	Random	0.92
	Female	1.29 (0.87, 1.89)	38%	0.17	Fixed	0.20

#### Test of heterogeneity, sensitivity analysis and publication bias

Significant heterogeneity between studies was observed in some comparisons; detailed data are shown in Table 5. Large heterogeneity was detected in some meta-analyses (*I* ^2^ = 59%, *P*_heterogeneity_ = 0.03). Then, random-effects models were used to evaluate combined ORs when necessary. Sensitivity analysis was performed both by sequential removal (statistics of study removal) of individual studies and by cumulative statistical analysis comparing subjects worldwide.

For the total population, sensitivity analyses indicated that the present study was the main source of heterogeneity. In addition, among male subjects, the combined ORs of the additive model and the dominant model were influenced by one study in HWE. The *P* values for the *Q*-tests became significant (*P*<0.05) after deletion of the study by Fava *et al.* Among female subjects, the combined ORs of the additive model, the recessive model and the homozygote contrast were influenced by the present study. The *P* values for the *Q*-tests became significant (*P*<0.05) after deletion of this study. Other combined ORs for the *CYP4A11* T8590C polymorphism were not influenced by the addition or removal of any individual study.

**Figure 2:**
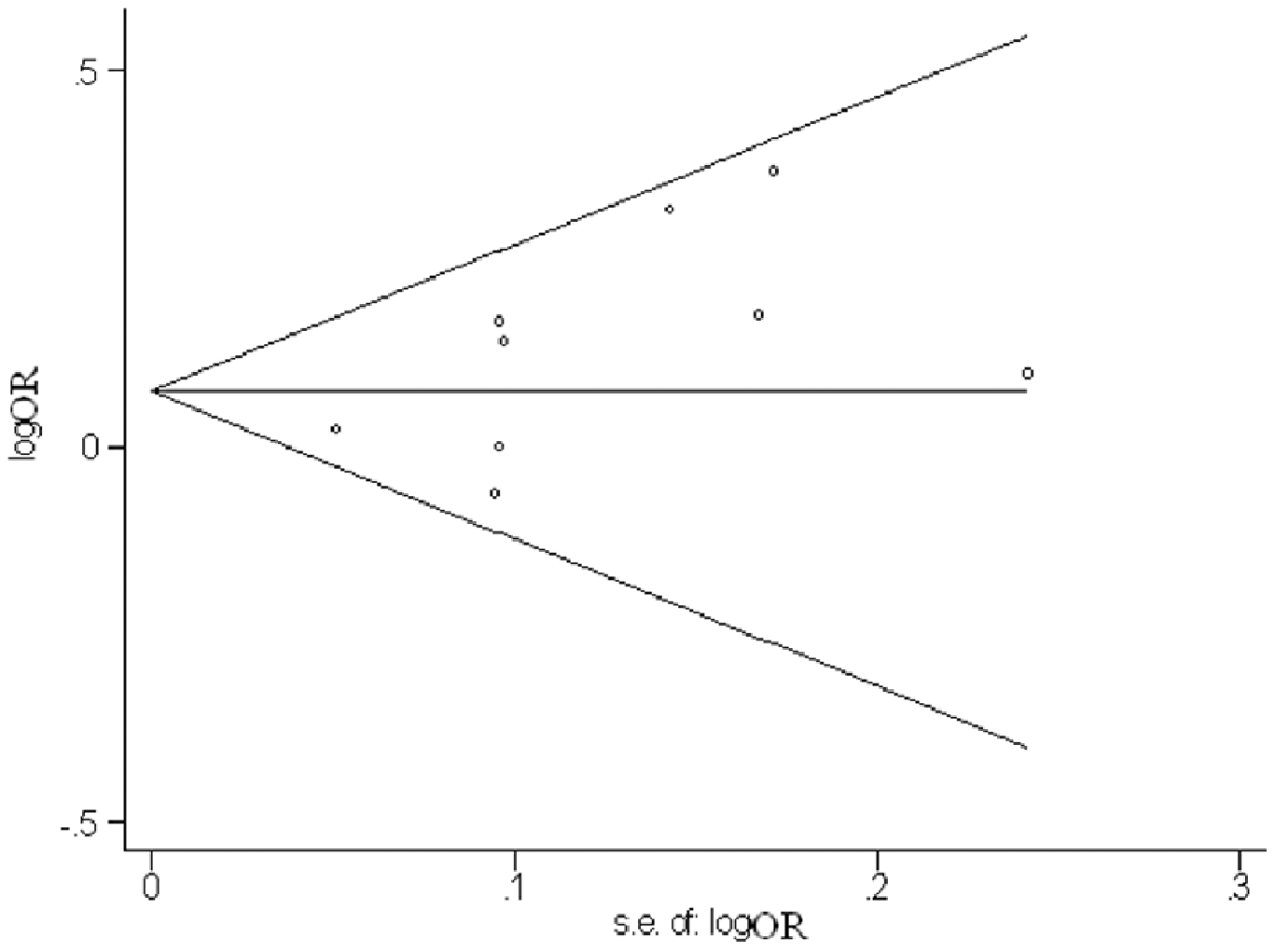
Funnel plot for studies showing the association between hypertension and the *CYP4A11* T8590C polymorphism using the additive model. The vertical and horizontal axes correspond to the odds ratios and confidence limits. OR, odds ratio; s.e., standard error.

A funnel plot and Egger’s test were performed to assess publication bias in this meta-analysis. The funnel plot showed no apparent evidence of publication bias (Figure 2). There was also no significant difference using Egger’s test in the allelic genetic model (*T* = 1.99, *P* = 0.09). The funnel plot and Egger’s test suggested that the selection of publications was an unlikely source of bias in this meta-analysis of the association between the *CYP4A11* T8590C polymorphism and essential hypertension.

## Discussion

The product 20-HETE, the monooxygenase derivative of arachidonic acid synthesized by CYP450 enzymes, is a candidate for the regulation of BP and hypertension. In humans, two members of the CYP4A subfamily have been detected in the kidney. *CYP4A11* has been reported to encode an enzyme that converts arachidonic acid to 20-HETE, primarily in the kidney [7]. Both animal and human studies indicate that the *CYP4A11* gene is a candidate causative gene for hypertension [22]. Recent studies have attempted to clarify the potential association between *CYP4A11* and essential hypertension. *CYP4A11* is known to be highly polymorphic, expanding the possibilities for studying the mechanism of action of this gene, especially as a risk factor for cardiovascular diseases [29]. A product of CYP4A11, 20-HETE, has been demonstrated to function in both a pro-hypertensive and an antihypertensive manner. In the renal arterioles, 20-HETE may act as a strong vasoconstrictor, whereas in renal tubules it may attenuate sodium transport and function as a natriuretic, antihypertensive substance. Ito *et al*. reported recently that CYP4A11 is highly expressed in human renal tubules and regulates 20-HETE, which is associated with the function of renal tubules and salt-sensitive hypertension [30]. Gainer *et al*. reported that the 8590T/C polymorphism of *CYP4A11* was functional, significantly reducing the catalytic activity of CYP4A11 enzyme and affecting the synthesis of 20-HETE [9]. Gainer *et al*. were also the first to demonstrate a positive association between 8590T/C and hypertension in whites as a loss-of-function variant. However, in 120 black subjects from the Tennessee cohort, there was no association between the 8590C allele and hypertension [9]. The association between the *CYP4A11* T8590C polymorphism and hypertension has also been studied by Mayer *et al*. [25]. It was reported that individuals with the CC genotype have higher systolic and diastolic BP. Fava *et al*. also reported that carriers of the C allele had a higher prevalence of hypertension in a prospective cohort including 6002 Swedish subjects [27]. However, another study demonstrated no significant difference in systolic or diastolic BP between the TC/CC genotype and the TT genotype of the *CYP4A11* gene in Australians [26]. Similarly, no significant association between the *CYP4A11* 8590T/C polymorphism and hypertension was observed by Sugimoto *et al*. in Japanese subjects [23]. The frequency of the (TC+TT) genotype was significantly higher in patients with hypertension than controls in a Japanese population [22]. In addition, 20-HETE has two opposing effects on BP (natriuretic and vasoconstrictor) and it is difficult to predict which effect will be more prominent.

In the present case-control study, no significant difference in the genotype frequency was observed between the cases and controls (*P* = 0.636) in the total population (Table 3). However, there were more males and smokers in the hypertensive population compared to the non-hypertensive population, which makes the results here more difficult to interpret. Even so, our results were in accordance with the findings of Sugimoto *et al*. and Ward *et al*. [23], [26], and were inconsistent with the findings of Gainer *et al.*, Mayer *et al.* and Williams *et al* [9], [24], [28]. Considering these inconsistent findings, we performed a meta-analysis of these studies to quantify the available data and generate a robust estimate of the effect of the T8590C polymorphism on hypertension. Meta-analysis has been proven to be a powerful method for analyzing a relatively large number of subjects.

In this meta-analysis, we analyzed the data from eight available case-control studies (including the present study) [9], [23]–[28]. The results concerning the role of *CYP4A11* polymorphisms in susceptibility to hypertension were conflicting. When all of the eligible studies were pooled for analysis, there was evidence for an association between the T8590C polymorphism and susceptibility to hypertension in various models. Moreover, on subgroup analysis by ethnicity, we found a significant association in Caucasians but not in Asians, suggesting genetic diversity between ethnicities. Therefore, it may be concluded that there is an association between the *CYP4A11* T8590C polymorphism and hypertension risk in the general population, especially among Caucasians. These results may provide conceptually novel approaches for study of the molecular basis of human hypertension, which could lead to new strategies for the early diagnosis and clinical management of this disease.

It is well established that the prevalence of hypertension and cardiovascular disease differs between men and women, the latter being protected from cardiovascular events until menopause; there is a rapid increase in the risk profile after menopause. Gene-specific effects have been indicated as responsible, but most genetic studies do not include sex as a variable in data analysis, and a recent meta-analysis pooling thousands of subjects of previous GWASs did not identify any sex-specific effect of genes associated with BP/hypertension. Experimental models suggest that enzymes involved in 20-HETE biosynthesis and metabolism have a role in the control of BP with a sex-specific effect [11]. In humans, previous studies have shown that the *CYP4A11* T8590C polymorphism appears to be a useful genetic marker of essential hypertension in men [18], [22]. In the present meta-analysis, although no gender-specific associations were found, after deletion of certain studies the *P* values became significant.

When interpreting these results, caution should be applied. Relatively large heterogeneity was present in the meta-analysis, perhaps due to the limited study sample size. A relatively limited number of studies and samples were analyzed, partly because the original studies had rather limited sample sets. Moreover, differences in the ethnicity and selection of controls, age distribution and lifestyle factors may have contributed to heterogeneity. Further large scale studies are needed to clarify whether the *CYP4A11* T8590C polymorphism is associated with hypertension risk in Asians or has a gender-specific effect.

## Conclusions

In summary, our meta-analysis suggests that the *CYP4A11* T8590C polymorphism is associated with risk of hypertension in the general population, especially in Caucasians.

## Supporting Information

Figure S1 **PRISMA flow diagram of the process of identifying and including articles for the systematic review.**
(DOC)

Checklist S1 **PRISMA checklist.**
(DOC)

## References

[1] Stokes J 3rd, Kannel WB, Wolf PA, D’Agostino RB, Cupples LA (1989) Blood pressure as a risk factor for cardiovascular disease. The Framingham Study–30 years of follow-up. Hypertension 13: I13–I18. 2535213 10.1161/01.hyp.13.5_suppl.i13

[2] Levy D, Ehret GB, Rice K, Verwoert GC, Launer LJ, et al (2009) Genome- wide association study of blood pressure and hypertension. Nat Genet 41: 677–687. 19430479 10.1038/ng.384 PMC2998712

[3] Newton-Cheh C, Johnson T, Gateva V, Tobin MD, Bochud M, et al (2009) Genome-wide association study identifies eight loci associated with blood pressure. Nat Genet 41: 666–676. 19430483 10.1038/ng.361 PMC2891673

[4] O’Donnell CJ, Lindpaintner K, Larson MG, Rao VS, Ordovas JM, et al (1998) Evidence for association and genetic linkage of the angiotensin-converting enzyme locus with hypertension and blood pressure in men but not women in the Framingham Heart Study. Circulation 97: 1766–1772. 9603529 10.1161/01.cir.97.18.1766

[5] Gainer JV, Brown NJ, Bachvarova M, Bastien L, Maltais I, et al (2000) Altered frequency of a promoter polymorphism of the kinin B2 receptor gene in hypertensive African-Americans. Am J Hypertens 13: 1268–1273. 11130770 10.1016/s0895-7061(00)01215-2

[6] Atwood LD, Kammerer CM, Samollow PB, Hixson JE, Shade RE, et al (1997) Linkage of essential hypertension to the angiotensinogen locus in Mexican Americans. Hypertension 30: 326–330. 9314412 10.1161/01.hyp.30.3.326

[7] Lasker JM, Chen WB, Wolf I, Bloswick BP, Wilson PD, et al (2000) Formation of 20-hydroxyeicosatetraenoic acid, a vasoactive and natriuretic eicosanoid, in human kidney. Role of Cyp4F2 and Cyp4A11. J Biol Chem 275: 4118–4126. 10660572 10.1074/jbc.275.6.4118

[8] Sarkis A, Lopez B, Roman RJ (2004) Role of 20-hydroxyeicosatetraenoic acid and epoxyeicosatrienoic acids in hypertension. Curr Opin Nephrol Hypertens 13: 205–214. 15202615 10.1097/00041552-200403000-00009

[9] Gainer JV, Bellamine A, Dawson EP, Womble KE, Grant SW, et al (2005) Functional variant of CYP4A11 20-hydroxyeicosatetraenoic acid synthase is associated with essential hypertension. Circulation 111: 63–69. 15611369 10.1161/01.CIR.0000151309.82473.59

[10] Fu Z, Ma Y, Yang Y, Zhang J, Xiang Y, et al (2008) Association between the SNPs of human CYP4A11 gene and essential hypertension. Journal of Xinjiang Medical University 31: 511–514.

[11] Fava C, Ricci M, Melander O, Minuz P (2012) Hypertension, cardiovascular risk and polymorphisms in genes controlling the cytochrome P450 pathway of arachidonic acid: A sex-specific relation? Prostaglandins Other Lipid Mediat 98: 75–85. 22173545 10.1016/j.prostaglandins.2011.11.007

[12] Wells GA, Shea B, O’Connell D, Peterson J, Welch V, et al The Newcastle-Ottawa Scale (NOS) for assessing the quality of nonrandomised studies in meta-analyses. Available: http://www.ohri.ca/programs/clinical_epidemiology/oxford. asp. Accessed 2012 Jul 6.

[13] Lau J, Ioannidis JP, Schmid CH (1997) Quantitative synthesis in systematic reviews. Ann Intern Med 127: 820–826. 9382404 10.7326/0003-4819-127-9-199711010-00008

[14] Capdevila JH (2007) Regulation of ion transport and blood pressure by cytochrome p450 monooxygenases. Curr Opin Nephrol Hypertens 16: 465–470. 17693763 10.1097/MNH.0b013e32827ab48c

[15] Capdevila JH, Falck JR, Imig JD (2007) Roles of the cytochrome P450 arachidonic acid monooxygenases in the control of systemic blood pressure and experimenta hypertension. Kidney Int 72: 683–689. 17597703 10.1038/sj.ki.5002394

[16] Zordoky BN, El-Kadi AO (2010) Effect of cytochrome P450 polymorphism on arachidonic acid metabolism and their impact on cardiovascular diseases. Pharmacol Ther 125: 446–463. 20093140 10.1016/j.pharmthera.2009.12.002

[17] Laffer CL, Gainer JV, Waterman MR, Capdevila JH, Laniado-Schwartzman M, et al (2008) The T8590C polymorphism of CYP4A11 and 20 hydroxyeicosatetraenoic acid in essential hypertension. Hypertension 51: 767–772. 18227405 10.1161/HYPERTENSIONAHA.107.102921 PMC2365894

[18] Gainer JV, Lipkowitz MS, Yu C, Waterman MR, Dawson EP, et a1 (2008) Association of a CYP4A11 variant and blood pressure in black men. J Am Soc Nephrol 19: 1606–1612. 18385420 10.1681/ASN.2008010063 PMC2488260

[19] Zhang R, Lu J, Hu C, Wang C, Yu W, et al (2011) A common polymorphism of CYP4A11 is associated with blood pressure in a Chinese population. J Hypertens Res 34: 645–648. 10.1038/hr.2011.8 21326303

[20] Ding H, Cui G, Zhang L, Xu Y, Bao X, et al (2010) Association of common variants of CYP4A11 and CYP4F2 with stroke in the Han Chinese population. Pharmacogenet Genomics 20: 187–194. 20130494 10.1097/FPC.0b013e328336eefe PMC3932492

[21] Fan C, He Q, Yu M, Zhong Y, Chen Z (2011) Association of Cyp4A11 gene polymorphisms with susceptibility to essential hypertension. Chin Prev Med 12: 662–665.

[22] Fu Z, Nakayama T, Sato N, Izumi Y, Kasamaki Y, et al (2008) A haplotype of the CYP4A11 gene associated with essential hypertension in Japanese men. J Hypertens 26: 453–461. 18300855 10.1097/HJH.0b013e3282f2f10c

[23] Sugimoto K, Akasaka H, Katsuya T, Node K, Fujisawa T, et al (2008) A Polymorphism Regulates CYP4A11 Transcriptional activity and is associated with hypertension in a Japanese Population. Hypertension 52: 1142–1148. 18936345 10.1161/HYPERTENSIONAHA.108.114082

[24] Mayer B, Lieb W, Götz A, König IR, Kauschen LF, et al (2006) Association of a functional polymorphism in the CYP4A11 gene with systolic blood pressure in survivors of myocardial infarction. J Hypertens 24: 1965–1970. 16957555 10.1097/01.hjh.0000244944.34546.8e

[25] Mayer B, Lieb W, Götz A, König IR, Aherrahrou Z, et al (2005) Association of the 8590T>C polymorphism of CYP4A11 with hypertension in the Monica Augsburg echocardiographic substudy. Hypertension 46: 766–771. 16144986 10.1161/01.HYP.0000182658.04299.15

[26] Ward NC, Tsai IJ, Barden A, van Bockxmeer FM, Puddey IB, et al (2008) A single nucleotide polymorphism in the CYP4F2 but not CYP4A11 gene is associated with increased 20-HETE excretion and blood pressure. Hypertension 51: 1393–1398. 18391101 10.1161/HYPERTENSIONAHA.107.104463

[27] Fava C, Montagnana M, Almgren P, Rosberg L, Lippi G, et al (2008) The V433M variant of the CYP4F2 is associated with ischemic stroke in male Swedes beyond its effect on blood pressure. Hypertension 52: 373–380. 18574070 10.1161/HYPERTENSIONAHA.108.114199

[28] Williams JS, Hopkins PN, Jeunemaitre X, Brown NJ (2011) CYP4A11 T8590C polymorphism, salt-sensitive hypertension, and renal blood flow. J Hypertens 29: 1913–1918. 21873888 10.1097/HJH.0b013e32834aa786 PMC3309034

[29] Cho BH, Park BL, Kim LH, Chung HS, Shin HD (2005) Highly polymorphic human CYP4A11 gene. J Hum Genet 50: 259–263. 15895287 10.1007/s10038-005-0245-9

[30] Ito O, Nakamura Y, Tan L, Ishizuka T, Sasaki Y, et al (2006) Expression of cytochrome P-450 4 enzymes in the kidney and liver: regulation by PPAR and species-difference between rat and human. Mol Cell Biochem 284: 141–148. 16552476 10.1007/s11010-005-9038-x

