# Supplementary material for: Association between the CYP4A11 T8590C Variant and Essential Hypertension: New Data from Han Chinese and a Meta-Analysis
Source: PLoS One. 2013 Nov 21;8(11):e80072. doi: 10.1371/journal.pone.0080072 (PMC3836999; doi:10.1371/journal.pone.0080072)
Supplement: Figure S1 — PRISMA flow diagram of the process of identifying and including articles for the systematic review. (DOC) [file pone.0080072.s001.doc]

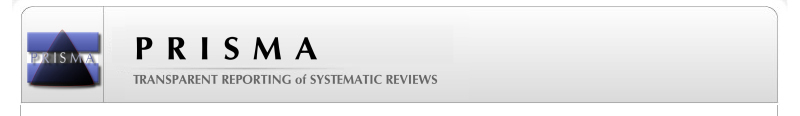
**PRISMA 2009 Flow Diagram**

**Screening**

**Included**

**Eligibility**

**Identification**

Records identified through database searching
(n = 14 )

Additional records identified through other sources
(n = 4 )

Records after duplicates removed
(n = 17)

Records screened
(n = 16)

Records excluded for reviews (n = 4 )

Full-text articles assessed for eligibility
(n = 12 )

Full-text articles excluded, with reasons (n = 4 )

Studies included in qualitative synthesis
(n = 8)

Studies included in quantitative synthesis (meta-analysis)
(n = 7 )

Full-text articles excluded,

for different criteria (n = 1 )
